# Sulfur starvation induces an Fe-replete response and attenuates virulence pathways in *Pseudomonas aeruginosa* PAO1

**DOI:** 10.1186/s12866-025-04442-1

**Published:** 2025-11-03

**Authors:** Chidozie G. Ugochukwu, Tonia S. Schwartz, Tonya N. Zeczycki, Douglas C. Goodwin, Holly R. Ellis

**Affiliations:** 1 Department of Chemistry and Biochemistry, Auburn University, Auburn, AL USA; 2 Department of Biological Sciences, Auburn University, Auburn, AL USA; 3 Department of Biochemistry and Molecular Biology, Brody School of Medicine, East Carolina University, Greenville, NC USA

**Keywords:** Pseudomonas aeruginosa, Sulfur, Oxidative stress, RNA-seq, Fe, Proteomics

## Abstract

**Background:**

Understanding bacterial responses to nutrient limitation is critical for developing targeted antimicrobial strategies. Sulfur starvation uniquely induces not only genes responsible for sulfur scavenging but also prominent antioxidant defenses. However, the biological rationale behind the simultaneous induction of antioxidants during sulfur limitation remains largely unexplored. Our study addresses this gap by integrating transcriptomic, proteomic, and targeted metabolomic data from *Pseudomonas aeruginosa* PAO1 grown under sulfur-free conditions.

**Results:**

As anticipated, transcripts and proteins involved in sulfur assimilation and metabolism—including members of the *msu*, *ssu*, and *cys* operons—were upregulated, along with key antioxidant enzymes such as Ohr, LsfA, and SodB. Unexpectedly, however, genes encoding iron uptake systems (pyoverdine, pyochelin, and heme metabolism operons) were markedly downregulated, while iron storage proteins (BfrB, Dps, and PA4880) were elevated, indicating an iron-replete metabolic state. Further targeted metabolic profiling and iron quantification assays confirmed reduced Fe acquisition and diminished extracellular levels of siderophore and phenazine metabolites. This shift in iron homeostasis correlated with the repression of multiple virulence factors regulated by Fur and PrrF, including quorum-sensing components, efflux pumps, and phenazine biosynthesis enzymes.

**Conclusion:**

Our integrative analysis reveals that sulfur starvation critically regulates iron homeostasis by linking reduced Fe uptake to the induction of antioxidant defenses. This iron-buffering response likely mitigates oxidative damage from unincorporated Fe, representing a protective metabolic adaptation. Additionally, the concurrent attenuation of virulence pathways suggests that targeting sulfur metabolism could disrupt iron-dependent virulence gene regulation, offering therapeutic insights into nutritional immunity strategies. Collectively, our findings uncover a novel sulfur-iron axis that plays a central role in oxidative stress management and pathogenicity modulation in bacteria.

**Graphical abstract:**

Using high-throughput RNA-sequencing and proteomics techniques, we identified genes and metabolites differentially expressed during sulfur starvation in *P. aeruginosa* grown in minimal media. Additional experiments using a range of biophysical techniques were used to quantify select metabolites and Fe. Overall, we found that sulfur starvation induced an Fe-replete response, characterized by the repression of Fe uptake pathways and the upregulation of Fe storage genes.

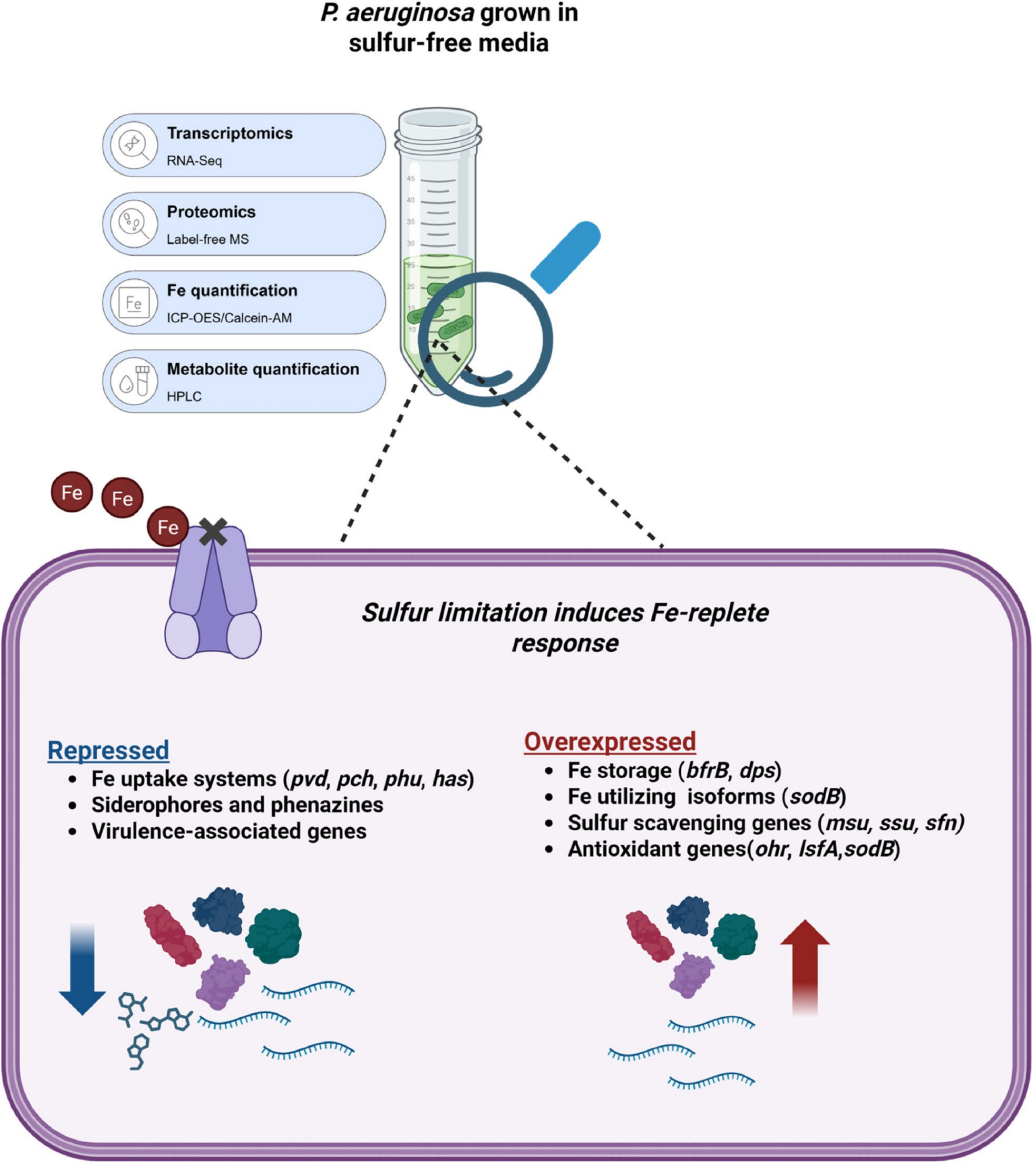

**Supplementary Information:**

The online version contains supplementary material available at 10.1186/s12866-025-04442-1.

## Background

*Pseudomonas aeruginosa* is an opportunistic pathogen that is a leading cause of morbidity in persons with weakened immune systems, cystic fibrosis (CF), burn wounds, and autoimmune diseases [1, 2]. *P. aeruginosa* is included on the ESKAPE pathogen list (an acronym for six bacteria of significant public health concern) owing to its virulence, resistance to most antimicrobial agents, and involvement in nosocomial infections [3, 4]. The virulence and multidrug-resistant capacity of *P. aeruginosa* derive from its relatively large (6 Mb) genome compared to most human bacterial pathogens [5]. This confers a degree of metabolic flexibility that enables it to thrive in aerobic, microaerobic (as seen in CF lungs), and anaerobic environments, adapting to utilizing the nutrients imposed by the prevailing niche microenvironment [6, 7].

The link between the metabolic versatility of *P. aeruginosa* and its pathogenic potential has driven investigations to understand how the organism adapts to different environmental conditions, antimicrobial assaults, and nutritional requirements [7–9]. Many of these studies have explored how *P. aeruginosa* adjusts to micronutrient deficiencies [10–13]. Other studies have investigated adaptive changes required for pathogenicity in acute and chronic infections [14–17]. Despite the critical role sulfur plays in cellular metabolism, there have been few studies [18, 19] that have investigated the effect of sulfur starvation in *P. aeruginosa*.

Sulfur is a bulk element required by cells for growth. In most cells, sulfur can constitute up to 1% of dry mass, where it is primarily incorporated into the sulfur-containing amino acids (cysteine and methionine) and cofactors, including thiamine, biotin, and coenzyme M [20]. Sulfur also plays a critical role in maintaining the cellular redox balance due to its ability to adopt a wide range of oxidation states (from − 2 to + 6) [21]. Transitions among these states cover a broad range of redox potentials and make sulfur a critical component in the biogenesis of redox-active compounds, including antioxidants such as glutathione, thioredoxin, glutaredoxins, and ferredoxins that contain reactive thiol groups or iron-sulfur clusters required in maintaining redox balance and various physiological functions in cells [22, 23].

Most bacteria prefer sulfate as their sulfur source, which is assimilated through a reduction pathway to form L-Cys [18, 19, 24]. However, in the absence of inorganic sulfur or when it is limiting, bacteria express a specific set of genes collectively termed sulfate starvation-induced (SSI) genes [18, 20] that are involved in the uptake and metabolism of organosulfur compounds. Interestingly, among the SSI proteins expressed during sulfur limitation are antioxidant genes required for scavenging reactive oxygen species (ROS), suggesting that the intracellular bacterial redox balance is disrupted when sulfur is limiting [18, 19, 25]. In *P. aeruginosa* PAO1, the oxidative stress response is primarily regulated by OxyR [26]. The oxidative stress response induces a set of proteins that reduce cytoplasmic H_2_O_2_ concentrations, decrease the intracellular iron pools, and repair H_2_O_2_-mediated damage [27].

Although previous studies have noted a correlation between sulfur starvation and antioxidant gene expression [18, 25], no investigation has provided a causal link between these two processes. Our overarching goal was to identify a plausible mechanism for the accompanying expression of antioxidant genes during sulfur starvation using omics and biophysical assays. Our study highlights the intertwined nature of sulfur and iron metabolism and underscores the importance of sulfur in maintaining cellular redox balance. An unexpected result from these studies is the downregulation of many virulence pathways of *P. aeruginosa* PAO1 during sulfur starvation. Clinical investigations targeting specific virulence pathways through nutritional immunity approaches can exploit this response to attenuate the organism’s pathogenic potential.

## Methods

### SFM preparation

Sulfur-free medium (SFM) was prepared as previously described [28]. Water for media preparation was double-deionized (Barnstead™ GenPure™, ThermoScientific, USA), and all glassware rinsed with 3 M HCl and distilled water before use. The final solution contained Tris-HCl (50 mM), sodium succinate (25 mM), NH_4_Cl (20 mM), KH_2_PO_4_ (0.5 mM), MgCl_2_ (0.5 mM), FeCl_3_ (2 µM), MnCl_2_ (1 µM), CaCl_2_ (1 µM), ZnCl_2_(1 µM), and 50 µg/mL of each proteinogenic amino acid except for cysteine and methionine. The medium was supplemented with 500 µM of sodium sulfate for the control group. The final solution was pH-adjusted (7.3) and filtered through 0.2 μm pore-size syringe strainers (VWR, USA).

### Growth assay

Colonies of *P. aeruginosa* PAO1 cells were cultured into 5 mL Luria-Bertani (LB) media overnight in a shaking incubator (37 °C, 220 rpm). Following this, 10 µL of bacterial suspension was used in inoculating a 48-well microtiter plate (Corning, Fisher Scientific, USA) containing either 990 µL of SFM (sulfur-starved test group) or SFM supplemented with 500 µM sulfate (control group) to a final dilution of 1:100. The plate was incubated in continuous shaking (250 rpm) mode in a Cytation™ 3 multimode plate reader (BioTek Instruments, Winooski, VT, USA) with the absorbance (A_600_) measured every 10 min for 14 h to monitor growth rate. Following this, exponential growth parameters were determined by plotting A_600_ against time (min). To identify the exponential growth phases for test and control cells, growth curves were modeled using the Gompertz and logistic growth models to determine exponential growth characteristics for both test and control cells using R software (v 4.4.1). The identified exponential phases were utilized for subsequent downstream analyses.

### Cell culture for omics investigation

*P. aeruginosa* PAO1 stock culture stored at −80 °C was plated on LB-agar and incubated overnight (~ 16 h) at 37 °C. To make biological replicates of *P. aeruginosa* cells, independent colonies from the LB-agar plate were cultured into tubes containing 4 mL of LB and grown overnight. The overnight cultures were then diluted 1:100 into either SFM (test group) or SFM supplemented with 500 µM sulfate (control group) and incubated at 37 °C in a shaking incubator until growth reached log-exponential phase (~ 5 h for test and 7 h for control). Cultures were split equally for RNA-seq and proteomics, pelleted at 5,000 × g for 5 min at 4 °C, flash-frozen on dry ice, and stored at − 80 °C until analysis.

### RNA-seq

We employed RNA-seq to quantify transcriptional changes resulting from sulfur starvation in *P. aeruginosa* cells. Three replicates of each test and control *P. aeruginosa* pellets were resuspended in 100 µL TE (10 mM Tris-HCl, 1 mM EDTA, pH 8) buffer containing 0.2 mg/mL lysozyme by vortexing. This was followed by incubating the mixture at 37 °C for 10 min to lyse the cells. Total RNA (> 200 nt) was then purified from the mixture using the Illustra™ RNAspin Mini RNA isolation kit (GE Healthcare, UK), following the manufacturer’s instructions. The quality and concentration of extracted RNA were assayed using agarose gel electrophoresis (1.25% agarose, 120 V, 35 min) and Nanodrop (ThermoFisher Scientific, USA). The RNA samples were then shipped to an NGS facility (Admera Health, NJ, USA) for sequencing. The integrity of the samples was assessed using an Agilent Bioanalyzer, with all six samples (three biological replicates per group) meeting the minimum requirement of an RNA Integrity Number (RIN) cutoff of seven.

#### Library preparation and sequencing

Samples underwent ribosomal RNA depletion using the QIAseq FastSelect − 5S/16S/23S Kits (Qiagen, USA), which enriched for mRNA. The rRNA-depleted samples were then processed with the NEBNext^®^ Ultra™ II RNA Library Prep Kit (NEB, Ipswich, MA, USA). Library quality was assessed using the Qubit^®^ dsDNA HS assay (Thermo Fisher, USA), High Sensitivity DNA ScreenTape (Agilent, Santa Clara, CA, USA), and qPCR with the KAPA SYBR^®^ FAST qPCR Master Mix Kit. Sequencing was performed on an Illumina Novaseq S4 platform (Illumina, San Diego, USA) using a 2 × 150 bp paired-end configuration, generating approximately 20 million reads per sample (10 million reads per end).

#### Bioinformatics and statistical analyses of reads

Downstream bioinformatics analyses were conducted on raw reads on the Galaxy platform [29] and the Alabama HPC clusters. Therein, FastQC (v 0.11.9) was employed for quality assessment of raw reads, Trimmomatic [30] (v 0.38.0) was used in removing low-quality reads(Phred score < 30), HISAT2 [31] (v 2.2.1) was utilized for read alignment and mapping to a reference *P. aeruginosa* PAO1 genome (GCA_000006765.1), and featureCounts [32] (v 2.0.1) employed for counting aligned reads. Differential expression analysis of gene counts was performed using the R Bioconductor package *limma* [33] (v 3.48.0). Differential expression analysis was conducted using the limma-voom method. Genes with counts per million (CPM) less than 0.5 in at least two samples were considered insignificant and filtered out, resulting in the removal of 19 out of 5,677 genes (0.33%). Library sizes were normalized using the Trimmed Mean of M-values (TMM) method. The voom function was used to transform count data to log_2_ CPM with associated precision weights, accommodating the mean-variance relationship inherent in RNA-seq data. Linear models testing the effect of sulfur starvation treatment relative to control were then fitted to the transformed data, and empirical Bayes moderation of the standard errors was performed using the eBayes function. Genes were deemed differentially expressed if they met the criteria of an absolute log_2_ FC ≥ 1 and an FDR-adjusted p-value ≤ 0.05. Positive differentially expressed genes are upregulated in sulfur starvation relative to the control, and negative differentially expressed genes are downregulated in sulfur starvation relative to the control.

Enrichment analysis was performed using the DAVID database, which aggregates annotation and enrichment information from various databases, including the Gene Ontology (GO) database. Enrichment p-values were calculated using Fisher’s Exact test. For our analyses, we corrected for multiple comparisons using the FDR method and a threshold of ≤ 0.05. Count and DE tables (Additional tables S1 and S5, respectively) were used in producing graphical outputs using GraphPad Prism and R-based packages (ggplot2 v 2.0.0).

### LC-MS/MS for label-free proteomics

Untargeted, label-free proteomics was utilized to understand the effect of sulfur starvation on the proteomic landscape of *P. aeruginosa* PAO1. Bacterial cell pellets, obtained as described in our cell culture protocol, were resuspended in urea lysis buffer (50 mM Tris, pH 8.0, 8 M urea, 40 mM NaCl, 2 mM MgCl_2_, and 1X HALT protease inhibitor) prior to sonication (4×, 30% amplitude, 10 s, 4 °C). Lysed cells were centrifuged at 10,000 x g for 10 min to isolate proteins. The total protein in the resulting supernatant was determined using bicinchoninic acid (BCA) assay with Pierce™ BCA protein assay kit (Thermo Scientific, Rockford, IL, USA), and 200 µg of each sample was diluted to a total volume of 200 µL using the lysis buffer without urea. Samples were reduced (5 mM DTT) at 32 °C for 30 min and then alkylated (15 mM iodoacetamide) in the dark for 30 min. Denatured, alkylated proteins were digested using trypsin (1:100 trypsin: protein) at 37 °C overnight after which the samples were acidified with 0.1% FA (final concentration). The resulting peptides were desalted using solid-phase extraction (Sep-Pak^®^ C18 Plus Short Cartridges, Waters) and eluted first with a solution of acidified (formic acid) ACN: H_2_O (25:75) followed immediately by a second elution step with 50:50 H_2_O: ACN + 0.1% FA. Samples were lyophilized to dryness overnight and then reconstituted in loading buffer (98:2 H_2_O: ACN, 0.1% FA) to a final concentration of 0.25 µg/µL. Peptide concentrations were verified using a colorimetric peptide assay (Pierce Quantitative Peptide Assay).

Purified peptides were analyzed by nanoLC-MS/MS using an UltiMate 3000 RSLCnano system coupled to a Q Exactive Plus Hybrid Orbitrap mass Spectrometer (Thermo Fisher Scientific) via nano-electrospray ionization. Peptides were separated using an effective linear gradient of 4–35% acetonitrile (0.1% formic acid) over 135 min. For data-dependent acquisition, MS spectra were acquired in positive mode. MS1 scans were performed at a resolution of 70,000 with an AGC target of 2 × 10^5^ ions and a maximum injection time of 100 ms. Data-dependent acquisition was used to collect MS2 spectra on the top 20 most abundant precursor ions with a charge > 1 and an isolation window of 1.5 m/z, a fixed first mass of 140 m/z, and a normalized collision energy for MS2 scans of 30. MS2 spectra were acquired at 17,500 resolution with a maximum injection time of 60 ms, an AGC target of 1 × 10^5^ and a dynamic exclusion of 30 s.

#### Proteomics data analysis

Fragpipe [34, 35] (v 20.0) was used for raw data analysis with default parameters for label-free quantification, match between runs (LFQ-MBR) workflow. Precursor ion m/z tolerance was ± 20 ppm with three missed cleavages for trypsin. Variable modifications included oxidation (+ 15.5995 Da on Met), carbamylation (+ 42.025 Da on Lys), and fixed modification carbamidomethyl (+ 57.025 Da on Cys). MS data were searched against the *P. aeruginosa* PAO1 reference proteome (Uniprot Proteome ID: UP000002438, accessed 7/2023) with modifications for common contaminants and decoy (50%) ions. Biological replicates for each experiment were identified in the search. The search results were filtered by a 1% false discovery rate (FDR) at the peptide level. Label-free quantification was carried out using IonQuant [36]. Match between runs FDR rate at the ion level was set to 1% for the top 500 runs. High confidence proteins (protein probability >0.95, at least 2 peptides per protein) were filtered and median-normalized.

Downstream statistical analysis, quality checks, and results visualization were conducted using a combination of R-based web tools (Fragpipe Analyst) [37], R-statistical packages (tidyverse suite v 1.3.2), and GraphPad Prism on output files from Fragpipe (*combined_protein*.tsv and *sample annotation* files). Differential expression analysis was performed using a protein-wise linear model combined with empirical Bayes using *limma* [33]. Perseus-type imputation [38] was used to establish relative abundance values for low-sampling proteins, and replaced them with random numbers drawn from a normal distribution with a mean of 1.8 standard deviations (SD) downshift and a width of 0.3 for each sample. The significance threshold was set at the adjusted *p*-value (Benjamini-Hochberg method) and absolute log_2_ fold change thresholds of ≤ 0.05 and ≥ 1, respectively. Result graphics were produced with ggplot2 and GraphPad Prism. Functional enrichment analysis was performed using Gene Ontology (GO) [39] and Kyoto Encyclopedia of Genes and Genomes (KEGG) [40] tool on STRINGApp [41] plugin of Cytoscape to identify enriched biological processes and pathways. The mass spectrometry proteomics data have been deposited in the ProteomeXchange Consortium [42] via the PRIDE partner [43] repository with the dataset identifier PXD062994.

### Fe inventory assay

#### Extracellular Fe quantification

To measure Fe levels in spent media using inductively coupled plasma optical emission spectroscopy (ICP-OES), *P. aeruginosa* cells from overnight LB cultures were diluted (1:100) into SFM or SFM with 500 µM sulfate and grown for 2.5, 5, and 7.5 h at 37 °C with shaking (220 rpm). Samples were harvested by centrifugation (5000 RCF, 4 °C, 10 min), and the supernatant was collected into 15 mL Falcon tubes after filtering through a 0.22 μm syringe filter (VWR, PA, USA).

Quantitation of Fe in the samples was carried out using an Avio 220 Max instrument (PerkinElmer, Waltham, MA), operated in argon mode. The instrument was calibrated before each analysis using a five-point (5–200 µg/L) Fe calibration curve prepared in 2% HNO_3_ (v: v). Fe was monitored at an emission of 238.204 nm, with one ppm of manganese (TruQ Multi-Element Standard, PerkinElmer, USA) used as an internal standard for axial torch alignment with a viewing height observation set at 15 mm. Plasma, auxiliary, and nebulizer gas flows were adjusted to 8, 0.2, and 0.7 L/min, respectively. Plasma power was set to 1500 W. During analysis, the peristaltic pump ran at 1 mL/min for sample uptake and 2 mL/min for flushing, with read delay, flush, and wash times set to 45, 90, and 120 s, respectively. An average of three reads per replicate (*N* ≥ 3) was obtained from the instrument and analyzed in GraphPad Prism for the statistical difference using an unpaired t-test with a significance threshold set at *p* ≤ 0.05. To account for differences in Fe abstraction from media due to cell density, Fe concentrations amongst groups were normalized using A_600_. The abstraction of Fe from spent media was calculated with the formula given below:

Relative [Fe]_spent media_ = ([Fe]_media_ – [Fe]_spent media_)/A_600_

#### Intracellular labile Fe^2+^

Intracellular labile Fe²⁺ levels were measured using Calcein-AM (ENZO Life Sciences, USA), a cell-permeable dye hydrolyzed by intracellular esterases to fluorescent calcein. Fluorescence is quenched upon Fe^2+^ binding, providing a measure of labile Fe. Cultures grown as above were sampled at 2.5, 5, and 7.5 h. Cell pellets (1 mL) were washed with PBS (pH 7.4), incubated with 5 µM Calcein-AM for 30 min in the dark at room temperature, washed twice, and resuspended in PBS. Fluorescence was measured using a Varioskan Flash multimode reader (ThermoFisher Scientific) at excitation/emission 495/515 nm, with 150 µL per sample in black 96-well plates. Untreated cells served as negative controls. Each condition was assayed in quadruplicate. Growth (OD₆₀₀) was monitored in parallel to normalize for cell density. Data were processed in R. Background fluorescence from blank wells was subtracted, and normalized fluorescence values (to OD₆₀₀) were averaged across replicates. Labile Fe levels at each time point were compared between conditions using Welch’s two-sample t-test in GraphPad Prism v10.4.1.

### Phenazine and Pyoverdine assay

High-Performance Liquid Chromatography (HPLC) was used to quantify phenazines and pyoverdine levels during sulfur stress. Filtered spent media, prepared as described previously, were used for the assays. Phenazine was separated using reversed-phase HPLC (Agilent 1100 series, USA) with a 20 µL sample injection, 1 mL/min solvent flow rate, and a C-18 column (Zorbax, Agilent, USA; 5 μm, 4.6 × 150 mm). A gradient mobile phase of 0.1% TFA in water (solvent A) and 0.1% TFA in acetonitrile (solvent B) was used, with a run time of 25 min. Absorption was monitored at 690 nm for pyocyanin (PYO) using a diode array detector under isothermal conditions (25 °C).

Pyoverdine was quantified using an HPLC instrument (Agilent 1260 Infinity II, CA, USA) equipped with variable wavelength and fluorescence detectors. Absorbance was monitored at 400 nm, and fluorescence emission at 460 nm (after exciting at 400 nm). Samples were manually injected (20 µL sample loop) with a 1 mL/min solvent flow rate, with separation achieved with a C-18 column (Pinnacle II, ThermoFisher, USA; 5 μm, 4.6 × 150 mm). The same solvents and gradients used for phenazine separation were employed but with a run time of 15 min. To account for variations in cell density between control and test groups, the obtained spectra peak areas were normalized using A_600_ for both metabolites.

## Results and discussion

### A. RNA-seq analyses of P. aeruginosa PAO1 under sulfur-limiting conditions

**Fig. 1:**
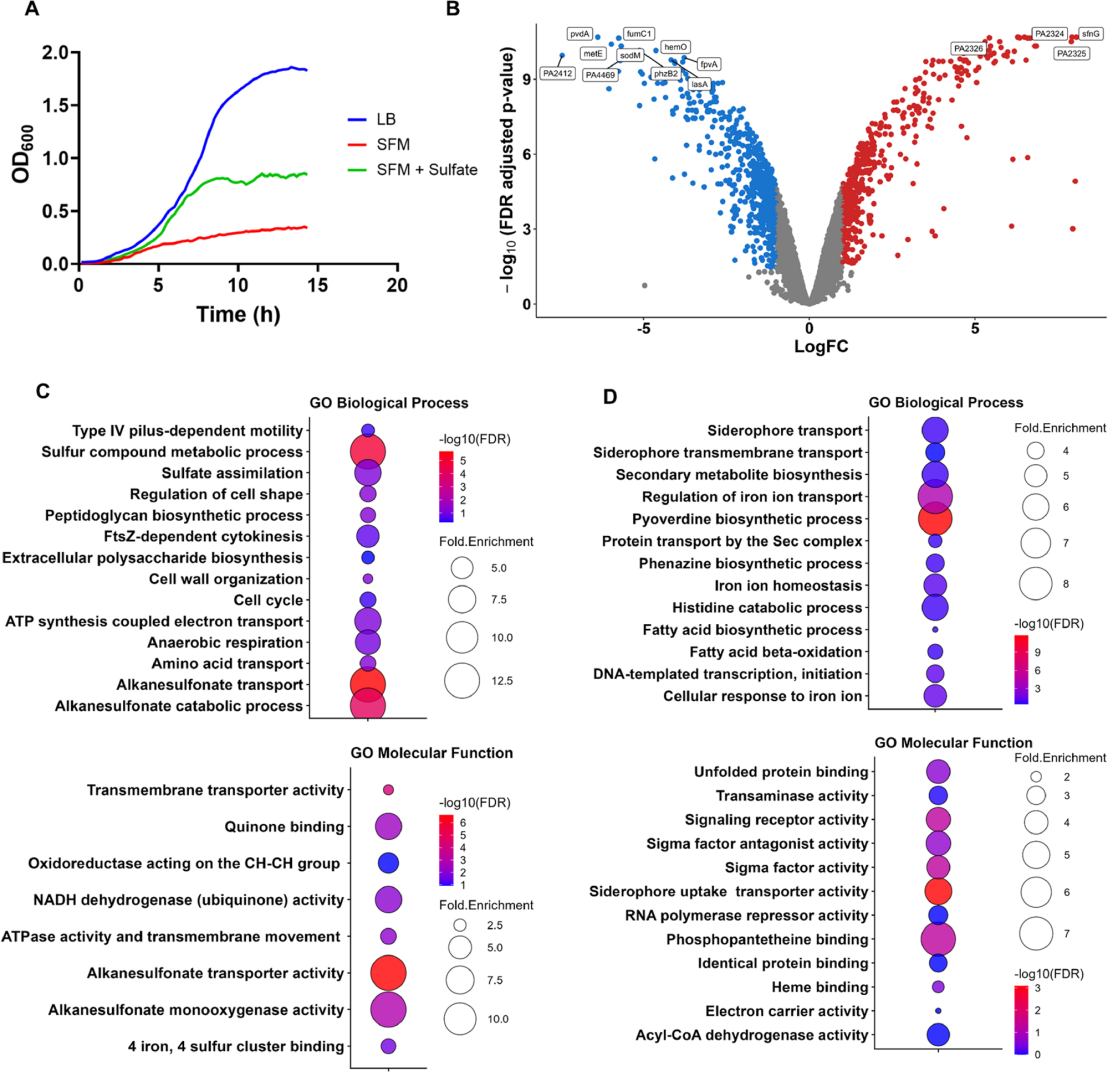
Sulfur starvation changes growth and transcription profiles of sulfur and iron genes. (**A**) Growth of *P. aeruginosa* PAO1 in minimal media lacking sulfur (SFM) or supplemented with 500 µM sulfate (SFM + sulfate) reveals significant growth depression under sulfur-deprived (test) conditions. Both conditions exhibit reduced growth compared to rich media (LB). (**B**) The volcano plot displays the log_2_ fold change (LogFC) between PAO1 cells grown without sulfur (test) and with 500 µM sulfate (control) on the x-axis, and the negative log_10_ of the false discovery rate (FDR)-corrected *p*-value on the y-axis. This analysis revealed 468 upregulated genes (red), 585 downregulated genes (blue), and 4,605 unchanged genes (grey) based on an absolute LogFC ≥ 1 and corrected p-value ≤ 0.05. The top 14 differentially expressed genes, ranked by both *p*-value and LogFC, are highlighted in the plot, with *pvdA* and *sfnG* being among the most downregulated and upregulated candidates, respectively, in the test versus control group. (**C**) Bubble plot displays Gene Ontology (Biological Process (top) and Molecular Function (bottom)) terms for upregulated genes, with bubble size and color representing fold enrichment and adjusted *p*-values for enriched process. Sulfur metabolism is the most enriched process in the list of upregulated genes based on a Fisher’s Exact Test for enrichment at a FDR of ≤ 0.05. (**D**) GO terms related to iron metabolism are the most enriched in the list of downregulated genes

Our study aimed to investigate the expression of genes, particularly antioxidant genes, during sulfur starvation in bacteria, using *P. aeruginosa* PAO1 as a model organism. We utilized functional genomics tools to obtain a comprehensive overview of gene expression changes associated with sulfur starvation. To fully evaluate the correlation between sulfur starvation and activation of reactive oxygen species (ROS) response genes, we first compared the growth of *P. aeruginosa* PAO1 in minimal medium lacking sulfur (test group) to the growth of PAO1 in media supplemented with 500 µM sulfate (control) (Fig. 1a). We modeled the growth using a Gompertz and logistic growth curves, respectively (Fig. S1a-b). This step was critical for determining the exponential growth phases for our samples, during which metabolic activity is maximal. We then conducted next-gen RNA sequencing (RNA-seq) on cells collected at the exponential growth phase.

Results from RNA-seq showed extensive changes in transcription patterns between sulfur-starvation and sulfate-supplemented conditions, with just over 1,000 genes differentially expressed (Fig. 1b; Additional Table S1), using an FDR-adjusted *p*-value threshold of ≤ 0.05 and an absolute log_2_ fold change (LogFC) of ≥ 1. Gene Ontology (GO) enrichment analysis of differentially expressed genes (DEGs) revealed that many of the genes with an increase in expression (fold change ≥ 10) were involved in sulfur and organosulfur compound uptake and metabolism (Fig. 1c; Table S2). These include genes of the *cys*, *ssu*, and *msu* operons that encode ABC transporters, FMN-dependent reductases/monooxygenases, dioxygenases, and sulfatases. Together, they form the sulfur starvation regulon required for scavenging and desulfurizing organosulfur compounds in diverse bacteria, including *P. aeruginosa* PAO1 [18, 44–46]. Additionally, we observed an upregulation of genes associated with the oxidative stress response.

Over 500 genes showed decreased expression, with approximately half of these genes directly or indirectly involved in iron (Fe) metabolism based on their GO terms (Fig. 1d; Additional Table S2). Although previous studies have reported regulatory connections between iron and sulfur metabolism in *P. aeruginosa* [47, 48], the direct impact of sulfur limitation on iron homeostasis has not been addressed. Our observations provide new insights into how defects in sulfur availability can influence both oxidative stress responses and iron regulation.

#### Effect of sulfur starvation conditions on sulfur acquisition and assimilation

**Fig. 2:**
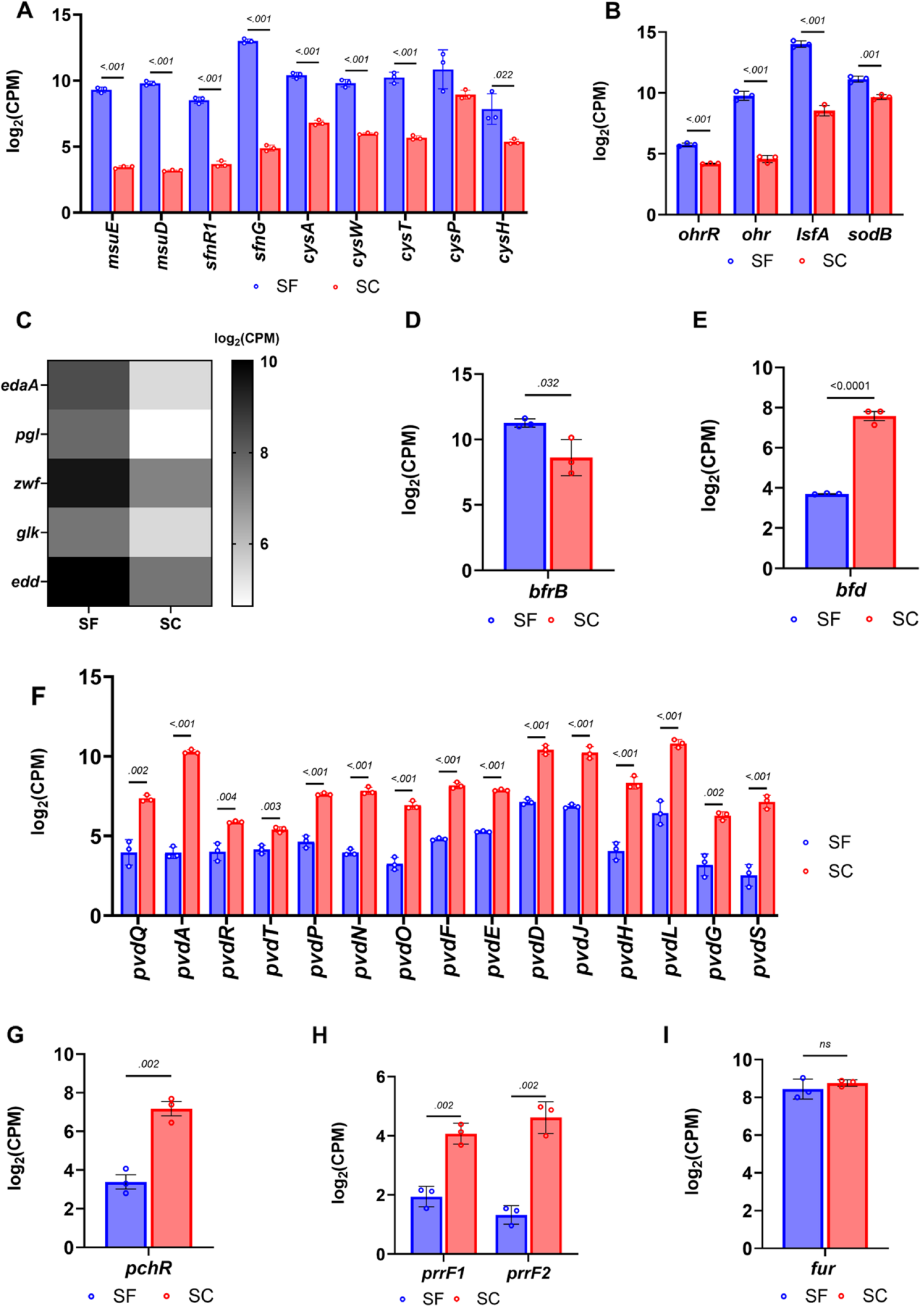
Differential expression of SSI, Fe-assimilation, and -storage genes (**A**). Overexpression of genes from the *cys* and *msu* operons and transcription factors from the *sfn* family involved in regulating genes associated with organosulfur uptake across the test (SF) and control group (SC). (**B**) Upregulated antioxidant genes, including *lsfA* (encoding a 1-cys peroxiredoxin), and *ohr* (and its transcription regulator *ohrR*), both involved in metabolizing organic hydroperoxides. (**C**) Heatmap displaying upregulation of genes in the pentose phosphate (PP) and Entner-Doudoroff (ED) pathways. (**D**) Upregulation of the *brfB* gene, involved in iron (Fe) storage, with a fold-change value of ~ 7.8. (**E**) Downregulation of the *bfd* transcript, which is involved in mobilizing Fe from the protein encoded by *brfB*. (**F**) Plot highlighting significant downregulation of *pvd* genes, which encode proteins required for the biosynthetic machinery of pyoverdine (PVD) siderophore uptake in PA. (**G**) Downregulation of the *pchR* transcript, the key regulator of genes involved in pyochelin synthesis and uptake. (**H) **Decreased expression of two small, non-coding RNAs, *prrF1* and *prrF2*. (**I**) *fur* transcript levels remained nearly identical across the test (SF) and control group (SC), reinforcing the role of Fur as an iron sensor. Statistical analyses were performed using GraphPad Prism. Gene expression levels were compared between SF and SC conditions using unpaired t-tests. Results are reported as mean ± SD. Multiple comparisons were corrected using FDR (Q = 5%). Statistical significance was defined as *p* ≤ 0.05

Prior studies on sulfur metabolism in pseudomonads have established the expression of sulfur-scavenging genes when challenged with alternative sulfur sources. Major gene clusters involved in organosulfur metabolism include the *msu*, *cys*, and *sfn* operons, which encode monooxygenases, ABC transporters, and transcriptional regulatory genes [18, 19, 49]. Notably, *sfnG*, which encodes an FMNH₂-dependent monooxygenase involved in utilizing alkanesulfonate, was among the most upregulated gene in response to sulfur starvation (fold change ~ 260). Other upregulated genes involved in sulfur scavenging include *PA0183* (*atsA*), an arylsulfatase (fold change >95); *msuD* and *msuE*, involved in methanesulfonate metabolism, both increased by over 50-fold during sulfur starvation (Fig. 2a). Although CysB is a putative regulator for the *msu* and *sfn* operons, there was only a marginal increase in *cysB* expression (*PA1754*) (fold change ~ 1.95x). Additional genes involved in cysteine metabolism and sulfur assimilation (*cysAWT*, *cysND*, *cysI*, *cysP*, and *cysH*) also regulated by CysB showed varying changes in expression levels with fold changes ranging from 2.5 to 25x (Fig. 2a). Similar increases in expression levels were observed for *P. aeruginosa* when provided with organosulfur compounds [19].

#### Oxidative stress response during sulfur starvation

Expression of genes involved in the oxidative stress response increases when alternative sulfur sources are used as a sulfur source [19]. A similar oxidative stress response was observed under sulfur starvation conditions as in the present work. One of the most upregulated antioxidant genes in our results was 1-Cys peroxiredoxin (*lsfA*) (>45 fold change) (Fig. 2b). The LsfA protein detoxifies ROS generated by NADPH oxidase produced by macrophages. This detoxification leads to increased virulence in *P. aeruginosa* strains [50]. The *ohr* gene (*PA2850*) involved in detoxifying different fatty acid hydroperoxides and peroxynitrite, was strongly upregulated in our results (>40x) [51]. A previous transcriptomics study also noted upregulation of the *ohr* gene with alternative sulfur sources [19].

Surprisingly, our results showed downregulation of a few antioxidant genes, including the catalases (*katA*, *katB*), and the alkyl hydroperoxide reductases (*ahpC*, *ahpF*, *ahpB*), all showing weak decreased expression at 2–3-fold (Additional Table S1). These antioxidant genes are regulated by OxyR, an H_2_O_2_ sensor in *P. aeruginosa* PAO1. Our RNA-seq data revealed that *oxyR* was unchanged (fold change = 1.5, FDR = 0.62). This may explain why the transcription of genes under its direct regulatory control remained unchanged or marginally downregulated. The increased expression of the *ohr* gene, whose regulation is independent of OxyR, suggests an oxidative stress mechanism that is potentially independent of H_2_O_2_ as a stressor. At the time point used to evaluate differential expression, lipid hydroperoxides could be generated in the absence of a sulfur source, leading to an increase in *ohr* expression.

Other genes involved in the ROS response that were differentially expressed include genes expressing superoxide dismutase (*sod*) (Fig. 2b). In *P. aeruginosa* PAO1, the principal SODs are SodA (Mn-SOD) and SodB (Fe-SOD). *sodB* showed >2-fold increase in expression, while *sodA* showed an ~ 50-fold decrease in expression in response to sulfur starvation. As will be discussed later, expression of the *sodB* gene is dependent on Fe-replete conditions and would show increased expression relative to *sodA* when iron is abundant. Additionally, genes involved in the pentose phosphate and Entner–Doudoroff pathways, including glucose-6-phosphate dehydrogenase (*zwf*) and phosphogluconate dehydratase(*edd*), were upregulated (Fig. 2c). The role of these proteins as a redox buffer during oxidative stress through the generation of NADPH and glutathione in *Pseudomonas* species has been previously reported [52, 53].

Overall, we observe a significant upregulation of antioxidant genes that have been typically known to be expressed during sulfur limitation or starvation in bacteria, with a minor exception—the slight downregulation of some catalases and alkyl hydroperoxide reductases.

#### Fe-replete conditions during sulfur starvation

Interestingly, we observed differential expression of genes involved in iron metabolism during sulfur starvation. This was characterized by a strong downregulation of genes involved in Fe uptake, accompanied by a pronounced upregulation of genes involved in Fe storage.

With respect to Fe uptake by *P. aeruginosa* PAO1, iron is scavenged via the use of siderophores, siderophore piracy (utilization of xenosiderophores), utilization of host heme proteins (cytochromes and hemoglobin through Phu and Has uptake systems), and direct internalization of ferrous iron using redox-active phenazines and the Feo system [14, 54]. *P. aeruginosa* PAO1 produces two siderophores: pyoverdine (PVD) and pyochelin (PCH). To utilize PVD, the major pyoverdine-class siderophore produced in *P. aeruginosa* PAO1, FpvA (*PA2398*) is required. FpvA is a TonB-dependent outer membrane receptor involved in iron uptake, ferrying PVD from the extracellular space into the periplasm [55]. In addition to FpvA, at least three other accessory proteins are involved in the uptake of PVD, including FpvG (*PA2403*), FpvH (*PA2404*), and FpvC (*PA2407*) [56]. The genes encoding these receptor proteins, except *fpvA*, are part of a large operon (*PA2403–PA2410*) involved in ferripyoverdine uptake [56]. Interestingly, we observed that the operon showed decreased expression, with log_2_ fold changes ranging from − 3.01 to − 3.7 (corresponding to 8- to 13-fold decreases) (Additional Table S1). Moreover, *fpvA* (*PA2398*), upstream of the operon, was also strongly downregulated. Separating the *fpvA* gene from the downstream *PA2403–PA2410* cluster is an operon encoding genes (*pvdG*, *pvdJ*, and *pvdL*) necessary for pyoverdine biosynthesis. These were also strongly downregulated, ranging from 10- to 30-fold decreases (Fig. 2f). Notably, the one of the genes demonstrating the largest decrease in expression, *PA2412* (>150-fold) (Fig. 1b), encodes an MbtH-like protein necessary for the production and secretion of pyoverdine at normal levels [57]. This further highlights the suppression of pyoverdine synthesis and accessory genes under sulfur limitation.

Like pyoverdines, the pyochelin (PCH) siderophore is secreted into the environment, where it binds ferric iron to form the PCH-Fe complex. The complex is internalized through the membrane transporters FptA and FptX [58]. The genes for the biosynthesis and transport of pyochelin are housed in two operons, which include the core biosynthetic clusters *pchDCBA* and *pchEFGHI* and their activator *pchR*, an AraC-type transcriptional regulator [59, 60]. PchR activates pyochelin biosynthesis and uptake genes when it binds the ferri-pyochelin complex under iron-limiting conditions [61]. This ligand-dependent activation, combined with Fur regulation of *pchR*, ensures that pyochelin production occurs only when iron is scarce and pyochelin is effectively able to acquire iron from the environment. In our results, *pchR* showed decreased expression (>10-fold decrease) (Fig. 2g), as did many of the genes in the two adjacent operons (*PA4218–PA4231*) involved in pyochelin synthesis and transport. The Fe(III)-pyochelin outer membrane receptor, including *fptA* (*PA4221)*, and the inner membrane permease *fptX* (*PA4218*), both showed a >2-fold decrease (Additional Table S1).

Other genes involved in Fe uptake that showed decreased expression in our RNA-seq data include those involved in xenosiderophore uptake, such as *femA*, *fiuA*, and *chtA* (fold changes ranging from 2- to 4-fold decrease) (Additional Table S1), and genes involved in ferrous Fe uptake, including *feoB* and *feoA* (7-fold and 17-fold decrease, respectively). Additionally, we observed decreased expression (>10-fold) of the *PA3407* gene encoding the heme acquisition protein HasAp. Along with other proteins encoded by the *has* operon, HasAp is implicated in virulence by mediating the uptake of host heme to scavenge iron during Fe-limiting conditions [62].

As noted earlier, we also observed the upregulation of genes involved in iron storage, reinforcing that sulfur limitation induces an Fe-replete response. We use the term “Fe-replete response” to describe the transcriptional and proteomic pattern normally associated with iron sufficiency (suppressed siderophore/uptake systems and increased iron-storage proteins), rather than an absolute increase in total cellular iron. The principal iron storage proteins in *P. aeruginosa* PAO1 are bacterioferritin BfrB (*PA3531*) and FtnA (*PA4235*) [10]. While the gene encoding BfrB showed increased differential expression (~ 7-fold increase) (Fig. 2d), there was no significant change in *ftnA*. BfrB is the major iron-storage protein in *P. aeruginosa* and associates with Bfd (*PA3530*; bacterioferritin-associated ferredoxin) to mobilize Fe^3+^ stored in BfrB when cytosolic iron levels drop below a certain threshold. This mobilization is facilitated by ferredoxin reductase (FprA) [10]. FprA reduces Fe^3+^ to the soluble ferrous form for cellular release. It is particularly striking that the genes for *bfd* and its accessory protein *fprA* were strongly downregulated in our results (~ 10-fold decrease) (Fig. 2e). Decreased *bfd* and *fprA* expression would prevent premature iron mobilization in cells already overburdened with unincorporated Fe.

The ferric uptake regulator Fur is a key iron-sensing transcriptional repressor that coordinates iron metabolism in *P. aeruginosa*. When intracellular Fe^2+^ is abundant, Fur binds Fe^2+^ as a co-repressor and dimerizes, enabling it to recognize the Fur box in target promoters. This represses expression of iron-uptake systems, including siderophore and heme transport genes, as well as the small RNAs PrrF1 and PrrF2. Under these conditions, iron uptake is shut down, and iron storage and utilization are favored to minimize cytoplasmic free iron-induced toxicity [63, 64]. In our RNA-seq data, *fur* transcript levels were unchanged during sulfur stress (Fig. 2i), which is consistent with Fur acting as an iron sensor rather than through changes in its own expression.

We observed strong downregulation (>4-fold) of the noncoding RNAs *prrF1* and *prrF2*, which normally repress nonessential iron-containing proteins under iron limitation [65, 66](Fig. 2h). Correspondingly, their downstream targets *sodB* and *bfrB* were upregulated, suggesting a shift toward increased iron utilization and storage during sulfur starvation.

In summary, we observed an overall response that includes the overexpression of genes involved in iron storage, a response typically seen in Fe-replete conditions. Conversely, there was a strong downregulation of genes involved in the various pathways used by *P. aeruginosa* to scavenge iron, including those for siderophores, heme metabolism, and ferrous iron uptake.

### B. Label-free proteomics support RNA-seq results

**Fig. 3:**
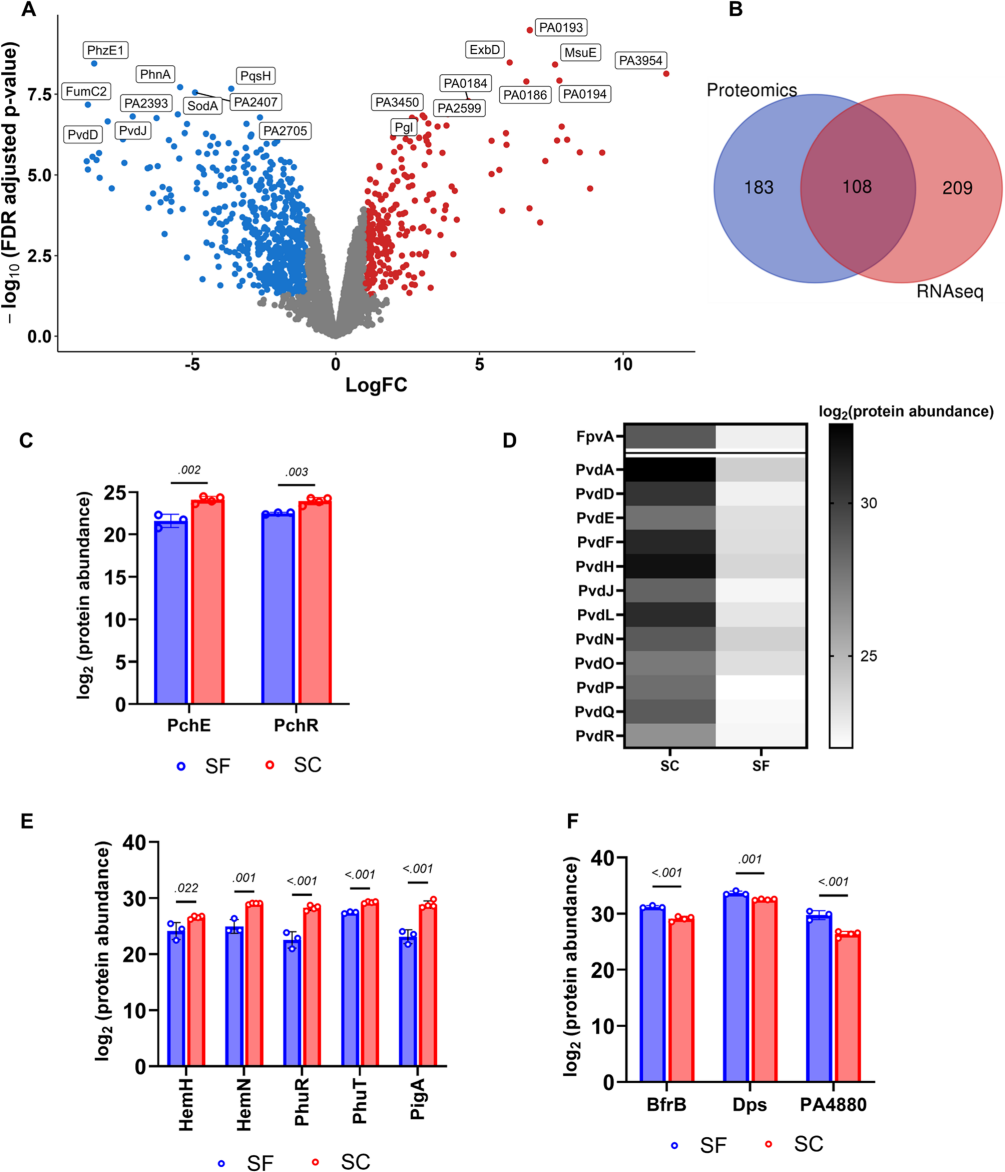
Fe uptake protein levels decrease as Fe storage protein levels increase (**A**) Volcano plot displays the LogFC between sulfur starvation (SF) and control sulfur-containing (SC) conditions on the x-axis, and the negative log_10_ of the false discovery rate (FDR)-corrected *p*-value on the y-axis. A significance cut-off of absolute LogFC ≥ 1 and corrected *p*-value ≤ 0.05 was applied. This analysis revealed 246 upregulated (red), 483 downregulated (blue), and 1,904 unchanged proteins (grey). (**B**) Proportions of features from RNA-seq and proteomics profiling that overlap when strongly differentially expressed features are compared (defined as absolute LogFC ≥ 2 and *p* ≤ 0.001). (**C) **Barchart showing downregulation of PchE and PchR, proteins involved in pyochelin metabolism, under SF conditions. (**D**) Heatmap displaying the downregulation of proteins involved in pyoverdine metabolism under SF conditions (**E**) Downregulation of proteins of the Phu and Has systems involved in heme uptake and metabolism under SF conditions. (**F**) Upregulation of bacterioferritin BfrB, Dps, and PA4480 proteins involved in iron storage under SF conditions. Results are reported as mean ± SD. Protein expression levels were compared between SF (*n* = 3) and SC (*n* = 4) conditions using unpaired t-tests, with FDR correction (Q = 5%) for multiple comparisons in GraphPad Prism. Significance was defined as *p* ≤ 0.05. Variances were evaluated using F-tests, and Welch’s correction was applied because of unequal sample sizes

To validate the findings from our RNA-seq experiments, we conducted label-free proteomics on *P. aeruginosa* PAO1 cultures grown with or without sulfate in sulfur-free media, consistent with our RNA-seq experiment. Our proteomics data identified 729 differentially abundant proteins compared to the 1,053 differentially expressed transcripts in our RNA-seq data, with the core enriched pathways being consistent between the two datasets (Fig. 3a-b).

As with our RNA-seq data, we observed an increase in the abundance of proteins involved in scavenging sulfur, with SfnG showing > 2,800-fold increase in the sulfur-starved test versus control. Other sulfur-scavenging proteins that were overexpressed include those from the *msu* (SfnR1 and MsuCDE) and *ssu* (SsuBDE) operons, with fold changes ranging from 2- to 470-fold (Additional Table S3). We also observed 2-to 6-fold increases in the abundances of permease proteins involved in taurine metabolism (TauAC), as well as marginal increases in proteins involved in cysteine metabolism, including CysW and the adjacent sulfate-binding protein (Sbp) (Additional Table S3).

Further, we observed increases in the abundances of the cognate proteins corresponding to the overexpressed antioxidant transcripts from our RNA-seq data. Some of the antioxidant proteins displaying increased abundances include Ohr (fold change ~ 2) and LsfA (8x) (Additional Table S3). However, SodB showed only a marginal increase that was not significant (fold change < 2, FDR > 0.05). Conversely, we observed a large decrease in SodA abundance under sulfur-starvation conditions (~ 45-fold), which may serve as a means of conserving manganese and ensuring that only iron-requiring isoforms of proteins are expressed during Fe-replete conditions. Consistent with our RNA-seq data, we also observed increased protein abundances for those in the pentose phosphate and Entner-Doudoroff pathways, including phosphogluconate aldolase (Eda) and 6-phosphogluconolactonase (Pgl), with fold changes of approximately 6-fold and 8.5-fold, respectively (Additional Table S3).

Interestingly, our proteomics data showed the same pattern of decreases in iron uptake proteins and pathways, as well as increases in proteins involved in iron storage, consistent with our RNA-seq results. This reinforces the notion of an Fe-replete condition induced by sulfur starvation. Moreover, decreases in protein abundances involved in siderophore-mediated (pyoverdine and pyochelin) uptake pathways were accompanied by a large (3.5- to 50-fold) decrease in proteins involved in heme metabolism (Fig. 3c-e); this includes proteins of the Phu and Has systems involved in heme uptake and metabolism. Conversely, the abundances of iron storage proteins, including the major *P. aeruginosa* PAO1 bacterioferritin BfrB, were increased (~ fold change 4x) (Fig. 3f). Furthermore, we observed increased expression of two proteins, PA0962 (Dps) and PA4880 (DpsL), which have been noted to serve as stores for ferric iron during iron-induced oxidative stress in *P. aeruginosa* PAO1 [67].

### C. Sulfur starvation induces downregulation of Iron-Linked virulence genes

An important observation from our transcriptomic and proteomic profiling is significant decrease in the abundance of gene products involved in virulence in *P. aeruginosa* PAO1, many of which have been linked to iron metabolism [13, 68–70]. Several genes encoding transcription factors, transport proteins, sigma factors, and small RNAs associated with virulence were differentially expressed in our results.

#### Multidrug resistance (MDR) efflux pumps

**Fig. 4:**
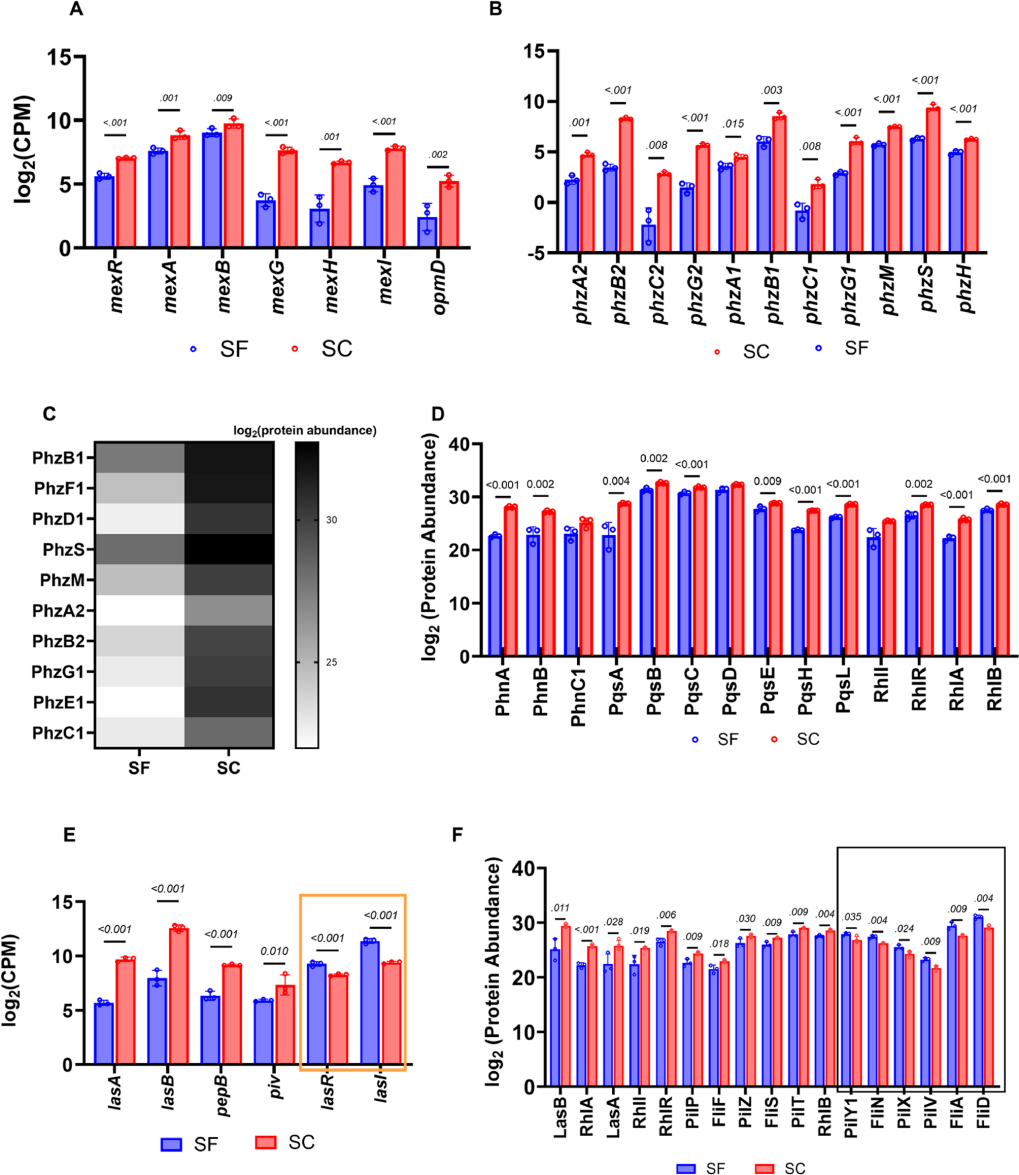
Repression of virulence gene products (**A**) Downregulation of genes of the *mex* operon involved in efflux pump biosynthesis in the sulfur starvation (SF) group. (**B**) Strong downregulation of genes in both phenazine biosynthetic clusters in test versus control. (**C**) Heatmap showing decreased abundances of phenazine proteins in sulfur starvation, reinforcing RNA-seq observations. (**D**) Barchart showing downregulation of proteins involved in quorum sensing, including those of the *phn*, *pqs*, and *rhl* operons. (**E**) Decreased expression of genes encoding virulence-associated proteases, including *lasA*, *lasB*, *pepB*, and *prpL*. Orange triangle highlights upregulation of *lasR* and *lasI*, thought to control the expression of some of the aforementioned proteases. **(F)** Chart showing a mixed response in proteins involved in biofilm formation, including the Las, Rhl, Fli, and Pil proteins. Box highlights proteins with observed increases in abundance under sulfur starvation conditions. Significance was defined as *p* ≤ 0.05, and results are reported as mean ± SD

Multidrug resistance (MDR) efflux pump transcripts, including *mexR* (PA0424), *mexA* (PA0425), *mexG* (PA4205), *mexH* (PA4206), *mexI* (PA4207), and *opmD* (PA4208), decreased with fold changes ranging from 2- to 13-fold (Fig. 4a) in our RNA-seq data. The *mexGHI-opmD* operon encodes an efflux pump involved in resistance to multiple antibiotics and is induced by endogenously produced phenazines [71]. The MexAB-OprM efflux pump system, which has a broad substrate range, is regulated by the MexR repressor located upstream of the operon [72, 73]. In our results, while *mexA* was downregulated (fold change >2), *mexB* and *oprM* remained relatively unchanged. Interestingly, *mexR* was also downregulated (fold change >2) in our RNA-seq data (Fig. 4a).

For our proteomic data, however, only MexH, OpmD, and MexT were significantly decreased in abundance, with corresponding fold changes of 14, 3, and 2.8, respectively (Additional Table S3). MexT, a LysR-type transcriptional regulator, acts as a transcriptional activator for another *P. aeruginosa* multidrug efflux pump system, MexEF-OprN, and also as a repressor for the outer membrane porin, OprD [74]. Moreover, it has been identified as a significant contributor to type III secretion (T3SS) systems in *P. aeruginosa*, which is important for virulence [75].

#### Phenazines and pyocyanin

Phenazines are aromatic, redox-active secondary metabolites produced predominantly by *Pseudomonas* species [76, 77] and have been implicated in virulence, iron assimilation, and primary energy metabolism in *P. aeruginosa* [78]. In *P. aeruginosa* PAO1, phenazines are initially synthesized as phenazine-1-carboxylic acid (PCA) via two independently acting, homologous operons: *phz1* (*phzA1B1C1D1E1F1G1*) and *phz2* (*phzA2B2C2D2E2F2G2*) that each encode seven enzymes. Three additional modifying enzymes-PhzH, PhzS, and PhzM-are required to convert core PCA into complex phenazine derivatives, including the blue phenazine pyocyanin [76].

Our RNA-seq results showed decreased expression of four of the seven genes in both phenazine clusters, with fold changes ranging from 2- to 80-fold (Fig. 4b), with more significant downregulation observed in the *phz2* cluster. Notably, the three remaining genes of the clusters (*phzD*, *phzE*, *phzF*) were absent from our results. This omission is potentially an artifact of our read-counting algorithm, which excludes multi-mapping reads to minimize potential over- or underestimation of paralogous genes. The high sequence identity of paralogous genes in both *phz* clusters (*phz1* and *phz2*) may lead to certain genes being entirely omitted. Additionally, the three genes encoding the tailoring enzymes required for complex phenazine biosynthesis (*phzH*, *phzS*, and *phzM*) were also downregulated with a fold change of at least two. Results from our proteomics experiments further validate this trend, showing a large decrease in the abundance of proteins belonging to both clusters of the phenazine biosynthetic operons, with fold changes ranging from 8- to 340-fold (Fig. 4c).

#### Quorum sensing and regulation of virulence proteases

*P. aeruginosa* PAO1 utilizes a complex quorum-sensing (QS) network to regulate the expression of numerous virulence factors, including secreted proteases, in response to environmental cues. The primary QS systems in *P. aeruginosa* PAO1 include the *Pseudomonas* quinolone signal (PQS), Las, and Rhl systems, each with distinct regulatory components and signaling molecules [79].

The PQS system, specifically 2-heptyl-3-hydroxy-4-quinolone, plays a pivotal role in QS and controls virulence factor expression across various infection models [80, 81]. PQS synthesis originates from anthranilate via the biosynthetic gene cluster *pqsABCDE*, an operon activated by the LysR-type transcriptional regulator MvfR [28]. Additionally, the small non-coding RNA PhrS, in conjunction with the RNA chaperone protein Hfq, enhances *mvfR* transcription through a base-pairing mechanism that activates *mvfR* mRNA translation [82]. In our RNA-seq results, genes within the *pqsABCDE* operon were downregulated at least two-fold under sulfur-starved conditions (Additional Table S1). While the expression of *mvfR* remained unchanged, both *phrS* and *hfq* exhibited a greater than 2-fold decrease, suggesting a potential mechanism for the downregulation of the *pqs* operon. This indicates that multiple regulatory factors may influence the transcription of *pqs* genes. Our proteomic data also showed similar decreases in protein abundances associated with the *pqs* operon (PqsABCDEHL) and the adjacent *phn* operon, which is involved in anthranilate biosynthesis, a precursor of PQS (Fig. 4d).

The Las QS system, consisting of LasR and LasI, is at the top of the QS hierarchy and controls the expression of various downstream genes, including those encoding the secreted proteases LasA and LasB [83]. LasA exhibits both elastolytic and staphylolytic activities, whereas LasB is primarily an elastase that can degrade host elastin, collagen, and immunoglobulins [84, 85]. These proteases play crucial roles in the pathogenicity of *P. aeruginosa* PAO1. Our RNA-seq data showed a greater than 2-fold increase in *lasR* and *lasI* transcripts under sulfur-starved conditions. Intriguingly, this upregulation was accompanied by a strong downregulation (~ 17- to 30-fold) of virulence genes thought to be under their control, including *lasA* and *lasB* (Fig. 4e). Similarly, proteomic data reflected a significant decrease in LasA and LasB protein abundances (Fig. 4f), despite no notable changes in LasR and LasI levels.

Other peptidases implicated in virulence in *P. aeruginosa* PAO1 include the extracellular proteases lysyl endopeptidase PrpL (also called PIV), involved in degrading lactoferrin, and the aminopeptidase PepB [86]. The genes coding for these proteolytic enzymes (*prpL* and *pepB*) were strongly downregulated in our RNA-seq results, with decreases as high as 30-fold. While our proteomics data showed a significant reduction in LasA and LasB protein abundances, PrpL levels remained unchanged and PepB was undetected. This discrepancy between transcriptomic and proteomic data for PrpL and PepB likely reflects methodological limits. Because only cell pellets were analyzed in the proteomics experiment, efficiently secreted proteins are underrepresented, reducing the reliability of transcriptome–proteome comparisons involving extracellular enzymes.

The Rhl QS system comprises the regulatory protein RhlR and the synthase RhlI, responsible for producing N-butanoyl-L-homoserine lactone (C4-HSL). The RhlAB proteins are involved in rhamnolipid production, which plays a crucial role in biofilm architecture and dispersion [16, 87]. Our RNA sequencing data revealed no significant changes in *rhlR* and *rhlI* expression levels under sulfur starvation. However, proteomic analyses indicated significant decreases (2- to 11-fold) in key Rhl system proteins, including RhlR, and RhlAB (Fig. 4d), highlighting a notable difference between transcriptomic and proteomic responses.

These findings underscore the potential impact of sulfur starvation on QS-regulated virulence factors at both transcriptomic and proteomic levels. The observed downregulation of key QS systems and their associated effectors suggests that sulfur limitation may attenuate the pathogenic potential of *P. aeruginosa* PAO1 by disrupting QS-mediated regulation. However, because QS activation is strongly dependent on cell density, we cannot rule out that the differences in *las*, *rhl*, and *pqs* expression reflect differences in growth yields between conditions rather than sulfur availability. Accordingly, the apparent sulfur-dependent regulation of QS genes and their downstream targets should be interpreted in the context of these limitations and warrant further interrogation to draw a definite conclusion.

#### Biofilm formation

*P. aeruginosa* PAO1’s ability to form biofilm is a significant determinant of persistence in CF patients. Studies have shown that iron metabolism affects biofilm formation [88, 89]. In these studies, iron starvation through growth in Fe-limited media, chelating Fe from the media using lactoferrin, or via mutation of genes involved in the synthesis of pyoverdines or uptake of ferric siderophores, including *pvdS*, *pvdA*, and *fpvA*, all led to increased twitching motility that prevents biofilm formation. In our RNA-seq result, we observed a mixed response in the levels of transcripts that have been implicated in biofilm formation. There was a 2-fold increase (Additional Table S1) in the expression of five genes (*pslF*,* pslH*,* pslI*,* pslJ*,* pslK*) of the *psl* operon involved in cell-cell interaction and biofilm formation. Some genes of the *pil* operon (*pilAY1Y2*) were also upregulated in the test cells compared to the control; they encode Type IV pili and facilitate initial attachment, surface motility (twitching), and biofilm formation maturation [90]. Other genes important in biofilm formation, including those of the *fli* (involved in flagellar biosynthesis and biofilm maturation) [91] and *alg* (responsible for alginate synthesis, a critical component of biofilm matrix) [92] operons were unchanged or undetected. Our proteomics data showed a similar trend; however, we observed decreases in Rhl proteins and a mixed response in the abundances of Fli and Pil proteins (Fig. 4f).

### D. Fe and Fe-associated metabolite levels in spent media corroborate an Fe-replete condition

**Fig. 5:**
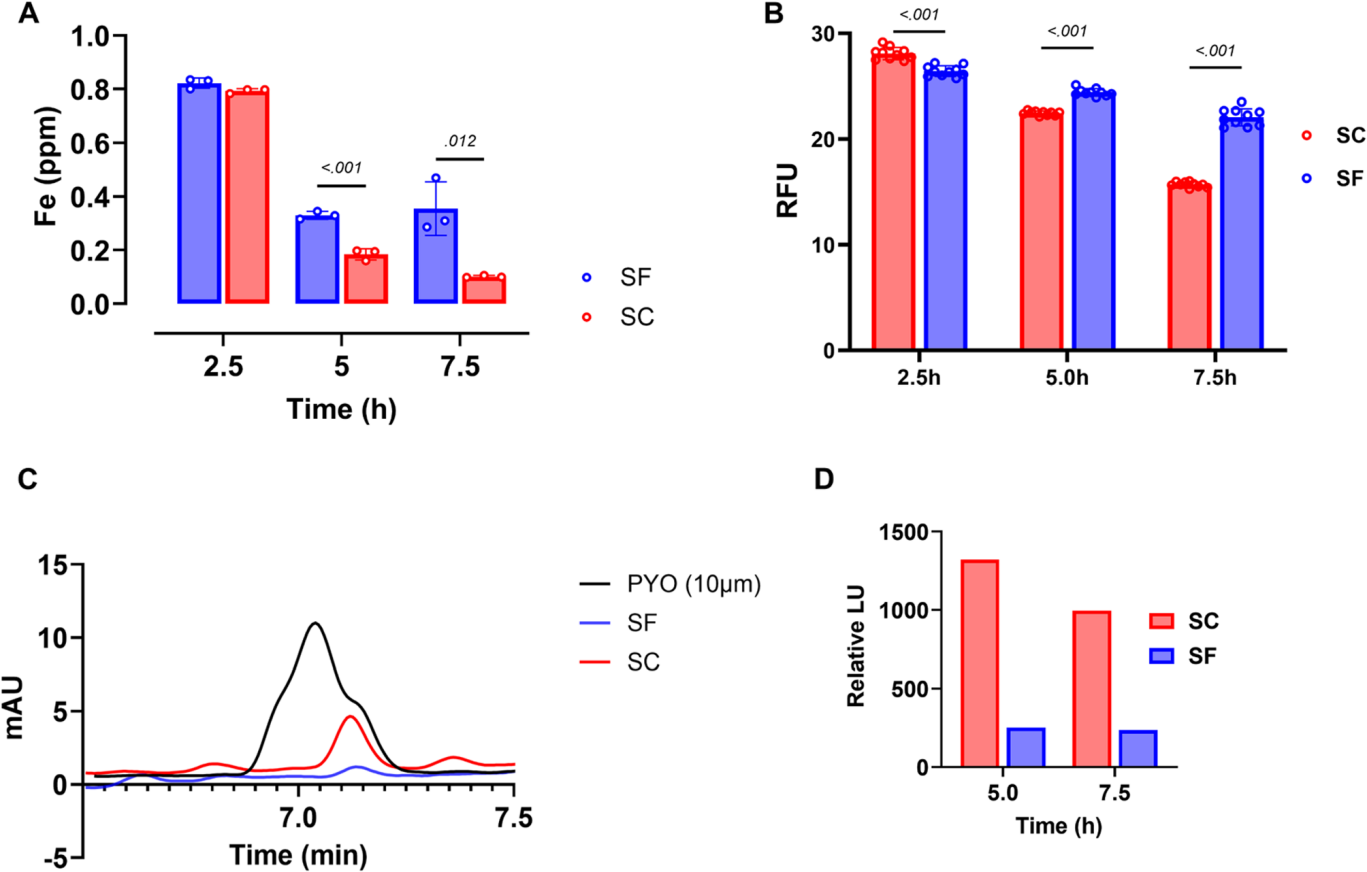
Changes in Fe and Fe-associated metabolites (**A) **Fe levels in spent media over time for sulfur starvation (SF) and control (SC) conditions. No significant difference was observed at the initial time point (2.5 h); however, at the later time points (5 h and 7.5 h), Fe levels remained higher in the SF group compared to the SC group, indicating reduced Fe uptake by the sulfur-starved cells. At time points 5 h and 7.5 h, Fe levels in the SF group remained constant, reinforcing the shutdown of Fe uptake pathways due to sulfur stress (**B**) Labile-Fe levels were monitored in the test compared to the control group using calcein-AM. The test cells exhibited a significantly higher level of unincorporated Fe than the control group at 2.5 h. However, at later time points (5 and 7.5 h), the control cells showed significantly more labile-Fe (**C**) HPLC traces display pyocyanin (PYO) levels in the SF and SC groups and a 10 μm PYO standard. Reduced PYO levels were observed in the sulfur-starved test compared to the sulfate-supplemented group. (**D**) Observed decrease in peak areas of pyoverdine under SF versus SC conditions at different time points. The SC group exhibited significantly higher peak areas compared to the SF group when normalized by A_600_, indicating greater pyoverdine production. Three independent assays were conducted and averaged. Unpaired t-test was employed to assess statistical significance using GraphPad Prism, with significance defined as *p* ≤ 0.05, where a significance test was appropriate

Having observed transcriptional signatures of an Fe-replete state during sulfur starvation, we next quantified extracellular Fe using ICP-OES. We hypothesized that sulfur-starved cells would import less Fe, leaving higher residual levels in the spent medium. Consistent with this, significantly more Fe remained in the sulfur-free cultures at 5 and 7.5 h compared to controls (Fig. 5a).

To assess intracellular Fe pools, we used the turn-off dye Calcein-AM, whose fluorescence is quenched by labile Fe. At 2.5 h, sulfur-starved cells showed reduced fluorescence, suggesting more labile Fe. However, at 5 and 7.5 h, fluorescence was higher in the test group, indicating less labile Fe than controls (Fig. 5b). We interpret this biphasic response as an initial accumulation of unincorporated Fe in sulfur-starved cells, followed by sequestration into storage proteins such as bacterioferritins, consistent with the observed induction of Fe storage genes. This likely reduces the labile Fe pool over time, preventing toxicity associated with excess Fe and Fenton chemistry.

Additionally, we investigated the potential decrease of siderophore production suggested by the -Omics investigations by assaying the levels of pyoverdine in spent media using high-performance liquid chromatography (HPLC). Elution times and peak shapes for pyoverdine were consistent across all samples (Additional Fig. 3), with the test group starved of sulfur having lower pyoverdine than the control (Fig. 5d). To confirm pyoverdine identity, an additional experiment was conducted using LB medium supplemented with the iron chelator DTPA, which induces pyoverdine production in *P. aeruginosa* PAO1. Overnight cultures grown in LB incubated with DTPA resulted in a 3-fold increase in the suspected pyoverdine peak (Additional Fig. 3). Full chromatograms for LB, SF, and SC samples are provided in the Additional file.

Given the observed decreases in phenazine gene products from our -Omics data, we conducted further experiments to measure the potential reduction in phenazine secretion by the sulfur-starved cells compared to the control. We measured pyocyanin levels, the most abundant form of phenazine, in the spent media of samples and found that the levels were lower in the sulfur-starved test compared to the control (Fig. 5c). This aligns with our observations from RNA-seq and proteomics results.

## Conclusion

Our study highlights a tight, mechanistic coupling between sulfur and iron metabolism in *P. aeruginosa* PAO1 and emphasizes sulfur’s central role in maintaining redox balance and iron homeostasis. Integrative omics demonstrate that sulfur starvation induces an iron-replete transcriptional and proteomic signature marked by the downregulation of iron uptake systems alongside upregulation of iron storage proteins. This response likely limits iron-mediated oxidative damage when Fe-S cluster assembly is impaired. Consistent with this interpretation, iron assays revealed reduced uptake from spent media and a time-dependent increase in intracellular iron storage. Together, these observations provide a coherent mechanistic explanation for the upregulation of antioxidant genes during sulfur limitation: impaired Fe-S biogenesis can increase the pool of free, unincorporated iron, promoting ROS formation through Fenton chemistry and thus necessitating an enhanced antioxidant response. The resulting homeostatic shift that sequesters labile iron plausibly mitigates iron toxicity. This mechanistic link addresses a gap in understanding the connection between sulfur limitation and antioxidant induction previously noted in the literature [19, 25, 93].

A notable, and initially unexpected, result was the marked depletion of multiple virulence-associated elements, including efflux pumps, phenazine biosynthesis, quorum-sensing systems, and secreted proteases, under sulfur starvation. These coordinated changes suggest that sulfur availability influences virulence potential in *P. aeruginosa*. We note that the observed transcriptional and proteomic shifts in these virulence factors are hypothesis-generating rather than definitive with respect to phenotype. Direct phenotypic validation of antibiotic susceptibility, biofilm formation, and in vivo virulence was beyond the scope of the present investigation and is required to confirm the consequences of sulfur restriction on virulence phenotypes.

Overall, our findings provide valuable insights into the adaptive mechanisms of *P. aeruginosa* PAO1 in response to environmental challenges. Understanding the regulatory networks governing sulfur and iron metabolism opens avenues for developing therapeutic strategies targeting these pathways. Modulating sulfur availability or disrupting sulfur metabolism could attenuate virulence and increase sensitivity to intervention with currently available antibiotics, offering multiple potential interventions against *P. aeruginosa* infections. By elucidating the mechanistic links between sulfur limitation, iron homeostasis, and oxidative stress response, our work advances the understanding of bacterial adaptation to nutrient stress. It underscores the importance of sulfur in bacterial physiology and pathogenicity, highlighting potential targets for antimicrobial development.

## Supplementary Information

Supplementary Material 1.

Supplementary Material 2.

Supplementary Material 3.

Supplementary Material 4.

Supplementary Material 5.

Supplementary Material 6.

## Data Availability

Raw RNA sequencing reads (fastq), count data, and metadata files have been deposited at the National Center for Biotechnology Information (NCBI) under Bioproject ID PRJNA1109844 with sequence read archive (SRA) accession numbers SRR28980933-SRR28980938. Proteomics files (.Raw files, fasta, and associated filtered protein files and metadata) have been deposited in the PRIDE database with an accession number (PXD062994). R scripts used for data processing, statistical analyses, and figure generation are available at the project’s GitHub repository: https://github.com/GoodwinEllisProject/Sulfur-Starvation-Response-in-P-aeruginosa.

## Abbreviations

CF: Cystic fibrosis
DEG: Differentially expressed genes
ECF: Extracytoplasmic function sigma factors
EN: Entner–Doudoroff pathway
ESKAPE: Enterococcus faecium, Staphylococcus aureus, Klebsiella pneumoniae, Acinetobacter baumannii, Pseudomonas aeruginosa, Enterobacter species
Fur: Ferric uptake regulator
LFQ: Label–free quantification
MBR: Match between runs
PCA: Phenazine–1–Carboxylic Acid
PP: Pentose phosphate
PVD: Pyoverdine
PYO: Pyocyanin
SC: Sulfur–containing
SF: Sulfur–free
SFM: Sulfur–free media
