# Supplementary material for: Sulfur starvation induces an Fe-replete response and attenuates virulence pathways in Pseudomonas aeruginosa PAO1
Source: BMC Microbiol. 2025 Nov 3;25:708. doi: 10.1186/s12866-025-04442-1 (PMC12581299; doi:10.1186/s12866-025-04442-1)
Supplement: Supplementary file 5 — Supplementary Material 5. [file 12866_2025_4442_MOESM5_ESM.pdf]

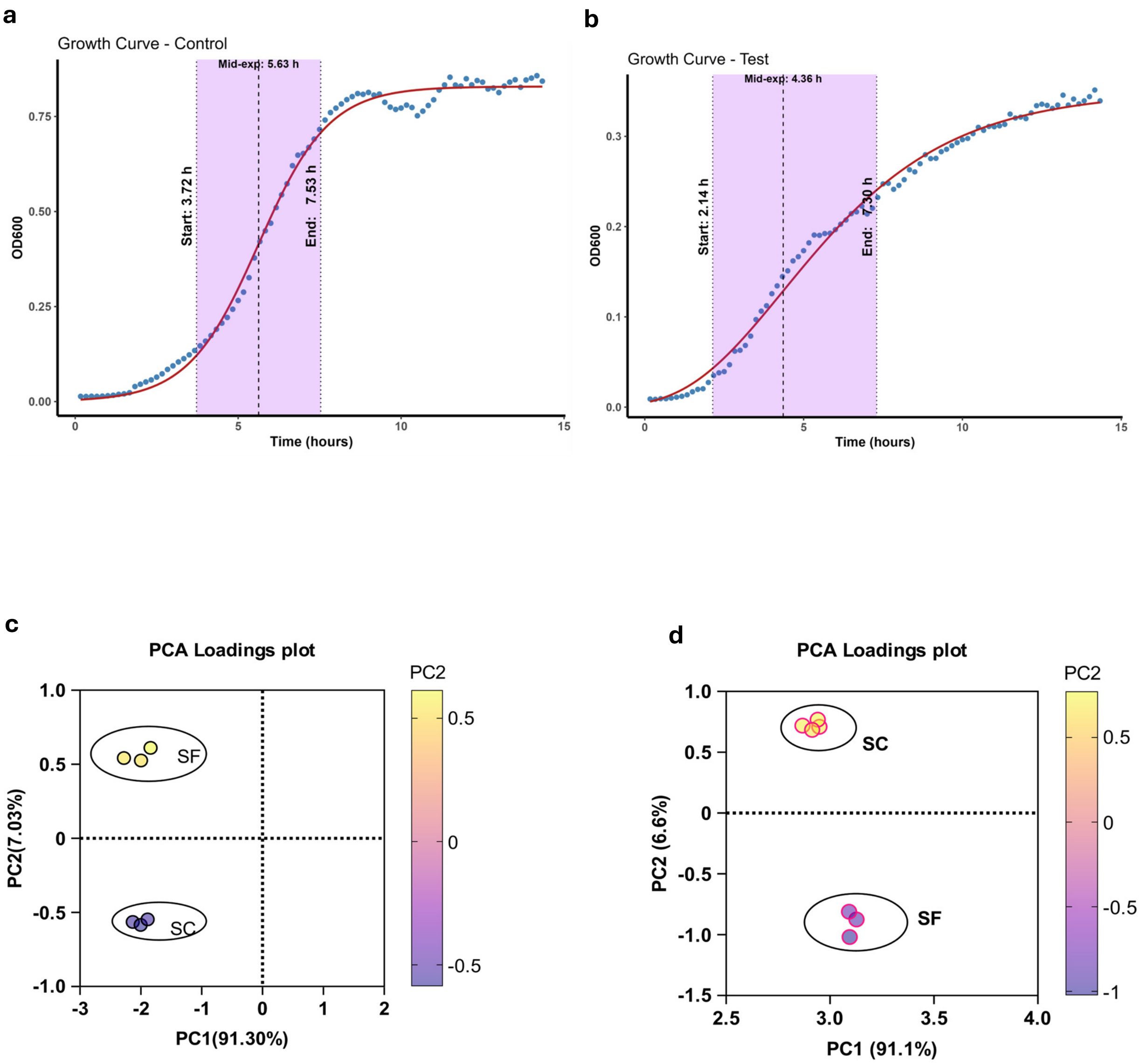

Supplementary Figure 1: Sulfur starvation alters *P. aeruginosa* growth and defines the exponential phase. Growth curves of *P. aeruginosa* PAO1 in sulfur-free minimal medium (SFM) or SFM supplemented with 500 µM sulfate (SFM + sulfate) both show marked growth depression relative to rich LB medium, with the sulfur-deprived condition exhibiting the slowest increase in OD_{600} **a-b)** To define the log-phase window, we fitted both Gompertz and logistic models to each dataset and selected the model minimizing the residual sum of squares (RSS). The **logistic** model best described the sulfate-supplemented (control) data, whereas the **Gompertz** model was optimal for sulfur-starved (test) cells. From these best-fit curves (red lines), the exponential phase (70% of maximal growth rate) spans 3.7–7.5 h for controls and 2.1–7.3 h for the test condition**. c-d)** Principal component analysis (PCA) result for RNA-seq and proteomics data, respectively. For RNA-seq, the first two components together explain ~98.3% of the total variance (PC1 = 91.3%, PC2 = 7.03%). Biological triplicates cluster tightly, indicating high reproducibility. A similar trend was observed with the proteomics data

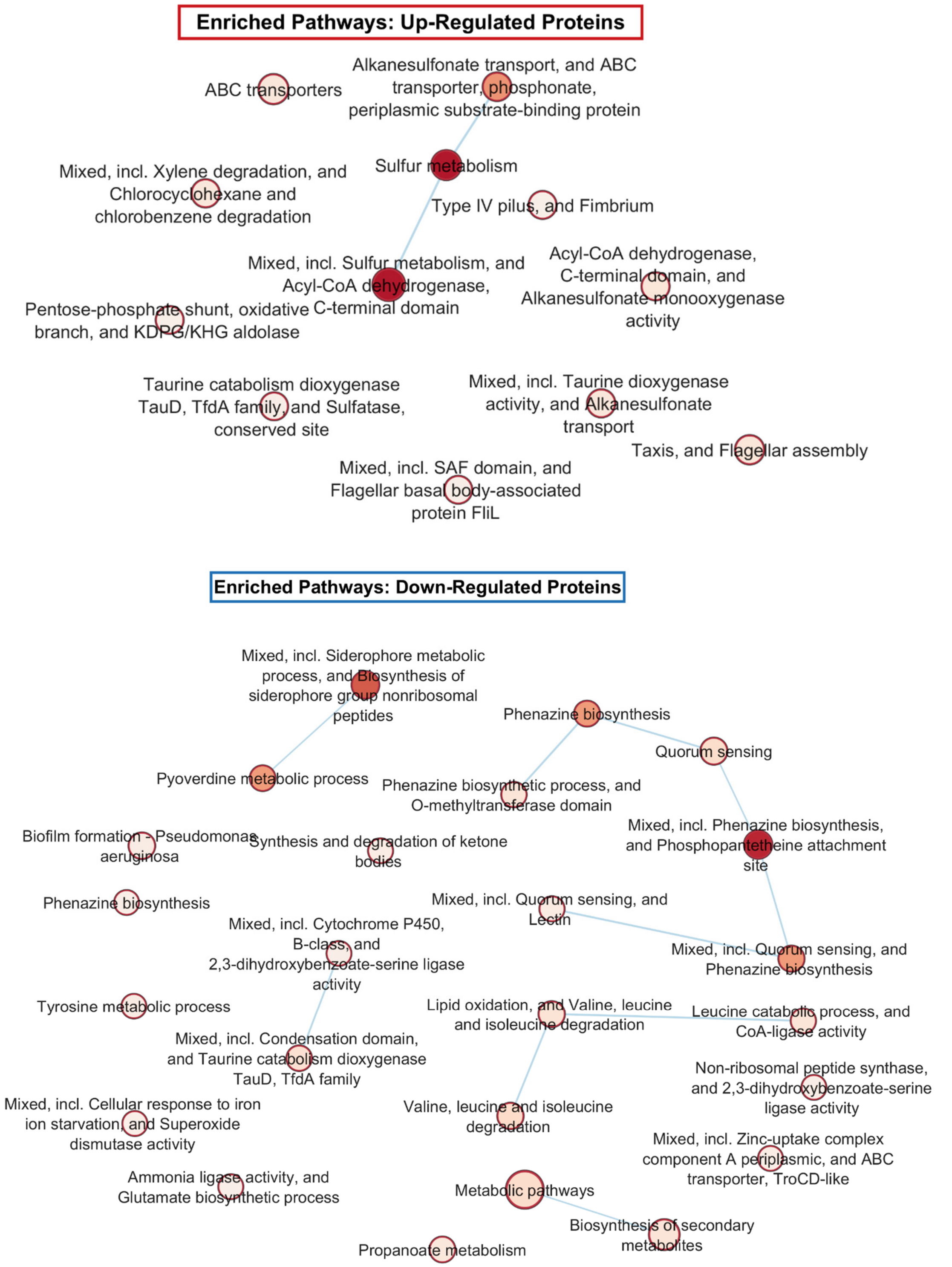

Supplementary Figure 2: Functional enrichment of differentially expressed proteins during sulfur starvation. Differentially expressed proteins were analyzed in Cytoscape using StringAPP and KEGG pathway enrichment, applying a redundancy cutoff of 0.5. EnrichmentMap was used to visualize significantly enriched terms (FDR $q < 0.05$). Each labeled node represents a pathway or biological process, with size proportional to the number of associated proteins and color indicating statistical significance (darker = lower q-value). Edges reflect shared proteins between terms. Siderophore, phenazine, and quorum-sensing pathways were the most enriched downregulated proteins, whereas proteins and pathways of sulfur metabolism were the most upregulated.

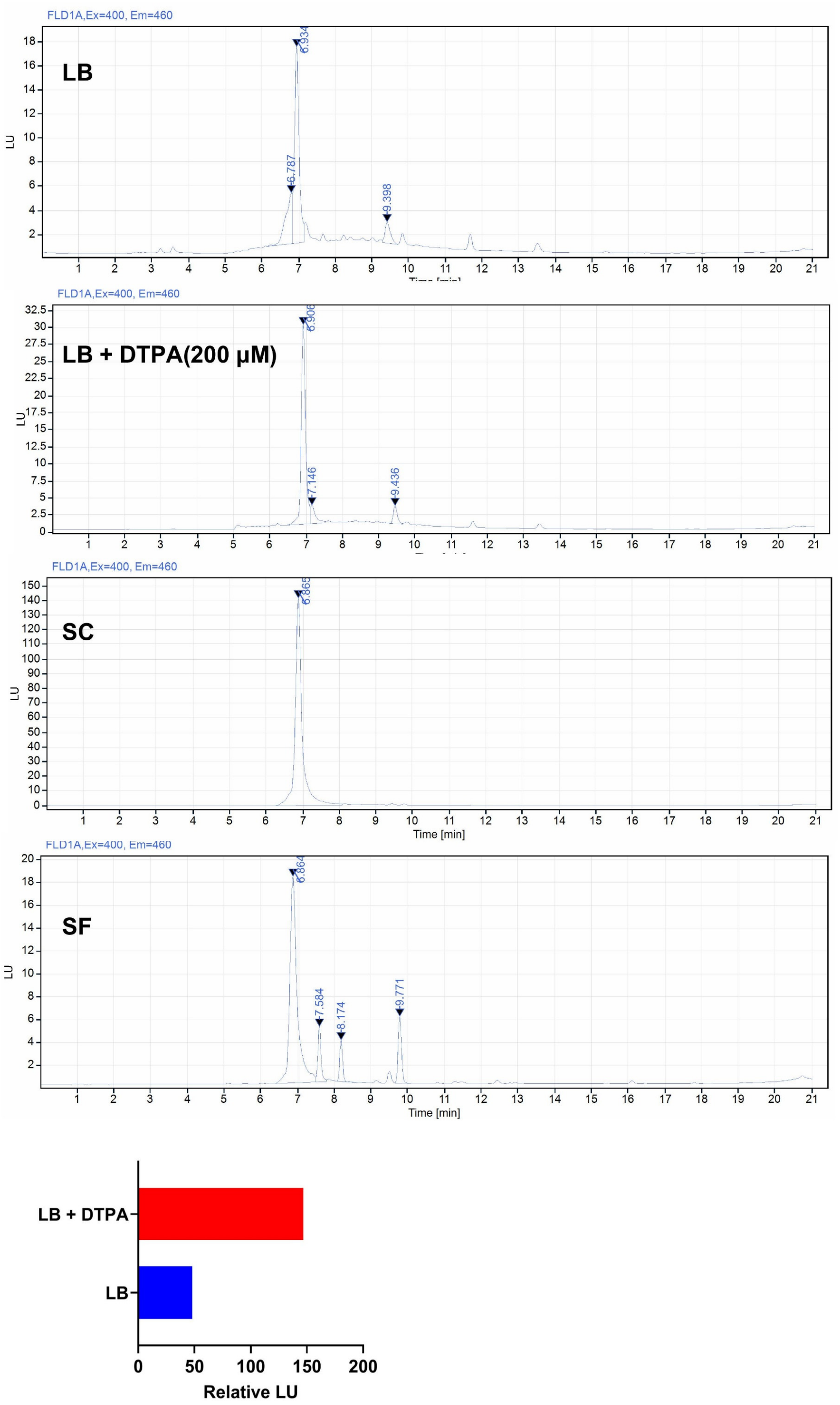

Supplementary Figure 3. HPLC traces for the relative quantification of pyoverdine between test (SF) and control(SC) treatments. The variation in pyoverdine levels in *P. aeruginosa* cells grown in rich media (LB) versus rich media supplemented with DTPA (an iron chelator known to stimulate pyoverdine secretion) is also presented. The bar plot displays the relative levels of pyoverdine in LB compared to LB + DTPA, normalized for cell density.
